# Maternal Influences and Intervention Strategies on the Development of Food Allergy in Offspring

**DOI:** 10.3389/fimmu.2022.817062

**Published:** 2022-02-23

**Authors:** Lefei Jiao, Chien-Wen Su, Tinglan Cao, Shasha Zheng, W. Allan Walker, Hai Ning Shi

**Affiliations:** ^1^ School of Marine Sciences, Ningbo University, Ningbo, China; ^2^ Mucosal Immunology and Biology Research Center, Massachusetts General Hospital and Harvard Medical School, Charlestown, MA, United States; ^3^ Laboratory for Lipid Medicine and Technology, Massachusetts General Hospital and Harvard Medical School, Charlestown, MA, United States; ^4^ Department of Nutrition, California Baptist University, Riverside, CA, United States

**Keywords:** maternal immunity, maternal microbiota, high-risk infants, food allergy, maternal effect, intervention strategies

## Abstract

Food allergies and other immune-mediated diseases have become serious health concerns amongst infants and children in developed and developing countries. The absence of available cures limits disease management to allergen avoidance and symptomatic treatments. Research has suggested that the presence of maternal food allergies may expose the offspring to genetic predisposition, making them more susceptible to allergen sensitization. The following review has focused on epidemiologic studies regarding maternal influences of proneness to develop food allergy in offspring. The search strategy was “food allergy OR maternal effects OR offspring OR prevention”. A systematically search from PubMed/MEDLINE, Science Direct and Google Scholar was conducted. Specifically, it discussed the effects of maternal immunity, microbiota, breastfeeding, genotype and allergy exposure on the development of food allergy in offspring. In addition, several commonly utilized prenatal and postpartum strategies to reduce food allergy proneness were presented, including early diagnosis of high-risk infants and various dietary interventions.

## Introduction

**Figure 1:**
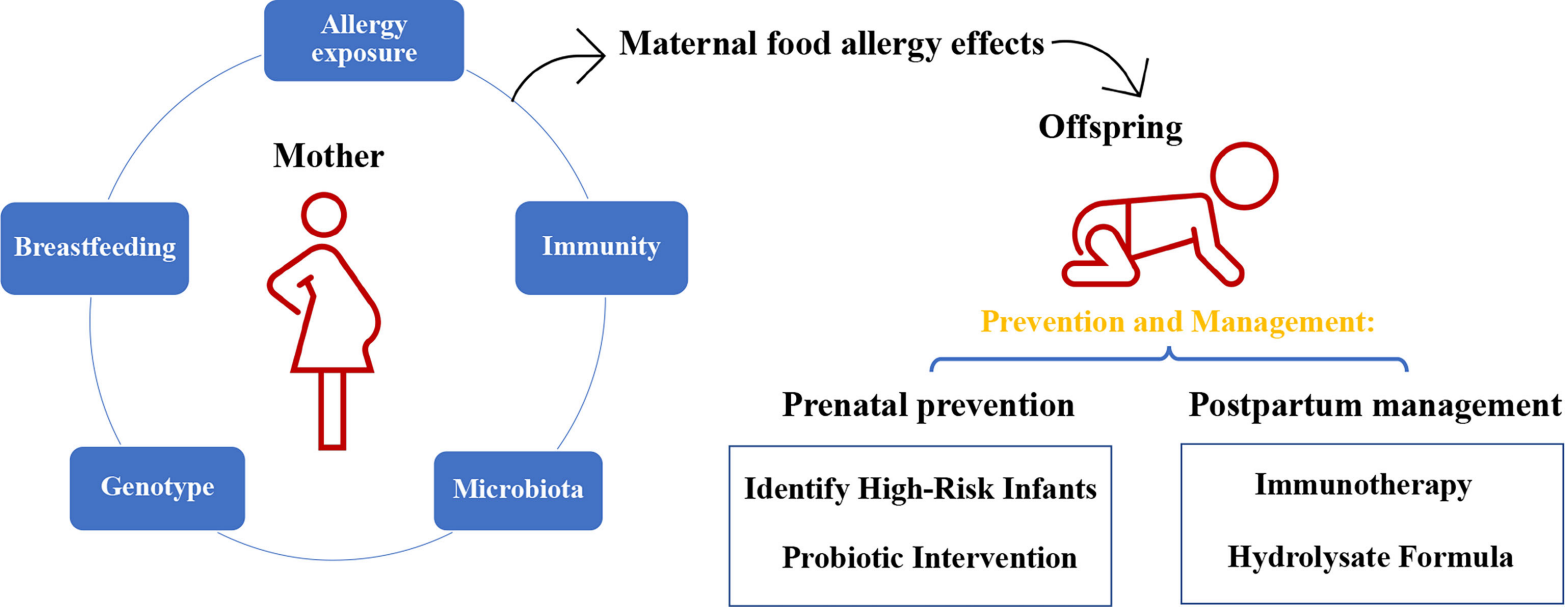
The impact of maternal food allergy on offspring and the method of prevention and management.

The term “allergy” describes an adverse immune response initiated by the host’s immune system upon exposure to a given substance that is generally harmless in the environment. Statistical analysis has shown that allergen-induced food allergies affect approximately 2.5% (ranging from 1% to 10%) of the world’s general population (1–3). Over the past several decades, food allergy has emerged unexpectedly as a “second wave” of the allergy epidemic, with an increasing number of infants and children afflicted with the disease from developing and developed countries worldwide (4–6). The prevalence of food-challenge-defined allergy to cow’s milk, egg, wheat, soy, peanut, tree nuts, fish, and shellfish was around 0.6%, 0.2%, 0.1%, 0.3%, 0.2%, 0.5%, 0.1% and 0.1% in Europe (according to the published paper from 2000-2012) (7). Research estimates that the food allergy currently affects 5–8% of the United States population, and the prevalence of food allergy in young children could grow as rapidly as 1% in a decade (8), while that of preschool children in developed and developing countries were as high as 10% (Australia) and 7% (China), respectively (proven by the oral food challenge test) (2). Throughout the years, accumulating evidence from epidemiological, *in vivo*, and *in vitro* experimentation has shed light on the possibility that infants with an atopic family history are genetically predisposed to allergies, especially those born from actively atopic mothers (9, 10). 12% of children with no family history of allergy, 30% to 50% of children with a single parental allergy and 60% to 80% of children with biparental allergies will develop allergic disease (11). In a population-based study of 5,276 one-year-old infants, children meeting the current definition of “high risk” for allergic disease (family members with a history of any allergic disease) showed an increase in food allergy compared to those with no family history of allergic disease (12). A possible explanation for this, supplementary to genetic similarity, is the individualized environment each mother provides for the infant during gestation and breastfeeding (13, 14). Therefore, a thorough understanding of the underlying mechanisms that mediate maternal influence to develop food allergy in children is critical to developing prevention and treatment strategies for allergic reactions and similar inflammatory diseases (**Figure 1**).

## Food Allergy

Typically, food allergens are either naturally occurring proteins or chemical haptens, which are small molecules that elicit immunological responses upon attachment to a larger carrier molecule and host immune recognition (15). Although some found in fruits and vegetables are only allergenic when the product is raw, most can persist through heat and acid treatment, such as during cooking and digestion. Out of the 170 potentially allergenic foods, only several are responsible for approximately 90% of all allergic reactions, with variations amongst different demographic groups and geographic locations (15, 16). Specifically, in the United States, food-specific immunoglobulin E (IgE) sensitization is commonly induced by milk, eggs, nuts, wheat, soy, and fish (7, 17). Symptoms of food allergy in babies can manifest as respiratory symptoms such as sneezing and runny nose, skin symptoms such as redness and hives, and intestinal symptoms such as constipation, flatulence and vomiting. The prevalence of food allergy is highest during early childhood, from infancy to toddler phase, and decreases slightly with age (18, 19). In cases where diversely sourced allergens share structural or sequential similarities, a process known as cross-reactivity can occur and elicit unexpected downstream effects. Still, the possibility of its occurrence depends highly on the type of food source where the allergen derives (20).

Based on the current understanding of the immunological mechanisms involved, the scientific community accepts that food allergies encompass IgE-mediated, non-IgE mediated, or a combination of both responses (21). IgE-mediated responses are the best-characterized amongst the few and involve the expression of antibodies known as IgE (22). Upon initial allergen exposure, plasma cells produce food-specific IgE from differentiated allergen-specific B lymphocytes that bind to tissue mast cells and blood basophils to induce allergic sensitization. During later allergen exposures, antigenic proteins can bind and cross-link with the surface-bound IgE, triggering the release of symptom-causing mediators, such as histamine and leukotrienes. Additionally, clinical presentations of the skin, gastrointestinal tract, and respiratory tract may appear as little as two hours of food ingestion (2).

On the other hand, non-IgE mediated, or cell-mediated reactions typically involve delayed-onset symptoms that occur approximately 4 to 28 hours after food ingestion (23). These reactions may exacerbate more severe conditions, such as protein-induced enterocolitis syndrome, allergic proctocolitis, or allergic contact dermatitis if left untreated. Despite the poor clinical and scientific definition of non-IgE mediated allergic reactions, evidence suggests T-cell involvement in its molecular mechanism.

## Influence of Maternal Immunity

From a genetic perspective, parental phenotype, precisely the maternal phenotype, is known to influence the offspring’s inheritance of atopic disease. With that in mind, a deeper understanding of the mechanisms underlying the influence of maternal immunity on the attenuation or exacerbation of offspring disease development is critical to the advancement of prevention strategies against atopic diseases.

A well-balanced maternal immune system will not only actively eliminate potentially harmful microbial stimuli, but also provide protective tolerance for the fetus. Infections during gestation profoundly influence the immunological environment provided by the mother, which then, in turn, affects offspring immunity through regulation of the Th1/Th2 cytokine profile and fetal-maternal immune transfer. *In utero*, this process occurs *via* the placenta and is subject to regulation by the placental barrier, which comprises of the tissue layers separating the maternal and fetal circulation systems. The neonatal Fc receptor (FcRn) on placental macrophages transports immunoglobulin G (IgG), the only antibody that can cross the placenta, *via* the syncytiotrophoblast (24–26). Among the four subtypes of IgG (IgG1, IgG2, IgG3, and IgG4), IgG4 associates closer with IgE during allergic reactions, given that allergen-specific IgG4/IgE ratios are significantly higher in allergy-tolerant individuals than allergy-prone individuals (27). The logic behind this observation is the fact that the non-atopic mother produces abundant IgG1 and IgG3 antibodies that are transported across the placenta to protect the fetus. In contrast, atopic mothers tend to produce IgE and IgG4 antibodies that are unable to cross the syncytiotrophoblast. Also, the newborn may be primed for allergic sensitization and develop early symptoms if fetal IgE is insufficiently downregulated by the lower levels of IgG1 and IgG3 produced by the mother.

Despite the lack of a complete understanding regarding the role of maternal allergen transfer in the development of offspring allergenicity, the most commonly postulated theory is that allergens can be transferred from the mother to fetus in the form of an IgG/allergen immune complex (28). In this case, fetal exposure to these antigens in the environment of the uterus may promote continuous tolerance against these specific food sources into the neonatal period. Evidence from both human and animal studies has suggested that allergic sensitization occurs during the prenatal period since the immunological environment within the uterus possibly affects the allergy development in the offspring. Several studies have also reported that a Th1 (T helper type 1 cell) response in the mother may offer the offspring protection against allergic reactions, demonstrated using a murine model and injecting non-allergic mothers with Th1 type immune inducers, such as dinitrochlorobenzene, LPS, and IFNγ (29–31). This study also shows how subjecting the offspring of allergic mothers to treatment with CpG oligonucleotides, a TLR9 agonist, and Th1-type stimulant, four days postnatal demonstrated protective effects against allergy development to suboptimal OVA (32). Considering the above, exposure of the allergic mother or offspring to a Th1 stimulus may help prevent an exaggerated immune response to a given allergen.

## Infant Immunity

The pregnancy stage offers the fetus an immunologic environment that is highly influenced by the maternal immune system. During the neonatal period, immune responses are more often allergic Th2 reactions rather than the protective Th1 response (33, 34). Despite the significant role of the mother, placental tissue also contains paternal antigens with a cellular composition different than their maternal counterparts. The Th1 responses elicited due to the cellular differences may put the developing baby at risk for possible rejection by the maternal immune system. The fetal environment bypasses this issue by developing a predominantly Th2 environment to suppress the mother’s Th1 response, stimulating the secretion of more Th2-type cytokines and less Th1-type cytokines. Upon maturation of the baby, the predominantly Th2 environment of the uterus switches to the protective Th1 type. However, this does not occur in atopic babies, where the Th2 response continues to predominate and sets the stage for allergen sensitization and subsequent allergic responses (35).

## Role of Breastfeeding

As an ideal, nutritional, immunologic, and physiologic nourishment, breast milk and the act of breastfeeding could enhance the newborn’s natural defenses to promote maturation of their immune system by increasing the levels of IgA, cytokines and probiotics (36). Within the human colostrum, 90% of all antibodies are secretory IgA that protects the infant’s mucosal surfaces until the infant produces adequate quantities of self IgA. Based on careful meta-analyses on evidence surrounding the role of breast milk in well-being promotion during infancy, The European Society of Pediatric Allergy and Clinical Immunology (ESPACI) and The European Society for Pediatric Gastroenterology Hepatology and Nutrition (ESPGHAN) strongly recommend exclusive breastfeeding for 4-6 months. Similarly, The American Academy of Pediatrics (AAP) recommends breastfeeding for at least four months and introducing complementary food items no earlier than 4-6 months for optimal allergy prevention (37).

Still, the preventative effects of breastfeeding against allergic disease development during infancy remain controversial since the nature of breastfeeding is highly influenced by maternal conditions during that period. The evidence suggests that breastfeeding can offer protective measures against food allergies when the mother is non-atopic. However, breastfeeding infants of atopic mothers may put them at higher risk of developing allergies (35), which can be explained by the higher levels of cytokines and chemokines and lower levels of transforming growth factor (TGF)-β1 in the breast milk of atopic mothers. Since TGF-β1 promotes food tolerance in the intestinal immune response, a healthy level present in the mother’s colostrum and breast milk would logically facilitate tolerance against various food allergens encountered by the infant from breastfeeding to ingestion of formula milk and solids. Nonetheless, conflicting studies still exist. Some researchers suggest that exclusive breastfeeding should be encouraged for at least 4 to 6 months in infants at both high and minimal risk for atopy and irrespective of a history of maternal allergy (38, 39).

Overall, conclusive evidence regarding either the protective or adverse effects of breastfeeding on food allergy onset is currently unavailable. Further research is required to ascertain whether breastfeeding has a positive or negative effect on the offspring’s development of atopic diseases.

## Allergen Exposure During Pregnancy and Lactation

The scientific community widely studied and debated the role of allergen exposure during pregnancy and lactation in allergy prevention. The method of allergen detection in the amniotic fluid and breast milk can provide insight into whether allergens from the mother’s diet can gain access to the developing baby and stimulate allergic sensitization (40, 41).

Previously, the American Academy of Pediatrics (2000) and the United Kingdom government (1998) recommended that atopic pregnant and breastfeeding mothers and their infants should avoid peanuts (42). However, no quantitative evidence suggests the fetal immune system is primed to respond to peanut allergens. Some authorities suggested the opposite: exposure to food antigens *in utero* may promote fetal tolerance (43) since the immune system can self-learn to distinguish between foreign or safe antigens to prevent initiating defensive action upon exposure to previously encountered items.

In 2008, the American Academy of Pediatrics withdrew its recommendations regarding the avoidance of specific food allergens (37), which was confirmed by the results of subsequent studies (44). Data taken from the Danish National Birth Cohort found that maternal peanut and tree nut intake during pregnancy played no significant role in the allergic outcomes of children at 18 months and seven years of age (45). Several recent studies also suggest the introduction of specific food allergens early on to enhance tolerance and prevent future allergic reactions. A study from 2014 reported that a higher maternal intake of peanut, milk, and wheat during early pregnancy could reduce the probability of mid-childhood allergy and asthma in the offspring (46). Similarly, the Learning Early about Peanut Allergy (LEAP) study assessed oral tolerance induction of peanut in high-risk children (with severe eczema, egg allergy, or both) aged between 4 and 11 months in the UK. Results showed that early introduction and regular ongoing consumption of peanut (average of 6 g of peanut protein a week) significantly decreased the frequency of peanut allergy among children already at considerable risk for developing this condition and, at the same time, modulated immune responses to peanuts (47). Even among mothers without peanut or tree (P/TN) allergy, higher pre-pregnancy consumption of P/TN was associated with a lower risk of P/TN allergy in their offspring (48), highlighting the beneficial effects of allergen exposure during pregnancy and lactation in food allergy expression. As reviewed above, maternal dietary exposure during pregnancy and lactation will unlikely contribute to food allergy development in children and may even help prevent them.

## Influence of Maternal Genotype

Although genetic risk factors would unlikely account for the recent spike in food allergy prevalence, they certainly play a role in one’s genetic predisposition for allergy and other atopic disease development. Today, researchers can consistently identify and replicate genetic loci associated with food allergies, such as the common STAT6, IL-10, CD14, IL-12 receptor b1, IL-4, TLR9, and FLG (49–53). Another large-scale genome-wide association study (GWAS) demonstrated a specific association between two allelic groups of the HLA-DQB1 gene (DQB1*02 and DQB1*06:03P) and peanut allergy (54). Still, we should note that previous genetic studies have mainly focused on individual genotypes without consideration for maternal influence during pregnancy and parental origins, which is why the aspect remains primarily unknown. A recent 2018 study represents the first attempt to examine maternal genotypic effects on food allergy by analyzing GWAS data generated from the Chicago Food Allergy Study. It was able to identify one single nucleotide polymorphism (rs4235235), located in a strand of non-coding RNA (LOC101927947), with significant maternal effect on any food allergy. However, its specific function is mostly unknown. Furthermore, the researchers observed three suggestive loci with maternal genetic effects: one regarding any food allergy (rs976078 located in the desert region of 13q31.1) and another two regarding an egg allergy (rs1343795 and rs4572450 located in the ZNF652 gene, whose genetic variants are associated with atopic dermatitis) (55).

Both host genetic susceptibility and environmental factors influence the complexity of food allergies (9). Studies regarding the complex gene-environment interaction effects on the intrauterine environment and, subsequently, fetal development requires further exploration.

## Maternal Microbiota

Despite the assumption that the fetal gut is sterile, recent evidence points to pregnancy as the beginning of bacterial exposure for the developing fetus upon identification of low bacterial levels in fetal membranes, meconium, amniotic fluid, and placenta (56–58). Such findings indicate the possibility of mother-to-fetus efflux of commensal bacteria through the placental barrier, breaking the sterility of the fetal gut. Considering this, the presence of bacteria or bacterial components during pregnancy may contribute to the immunological stimulatory, protective, or depletory effects for the neonate. Several researchers used animal models to demonstrate further the influence of maternal gut microbiota on offspring immunity and intestine microbiota (59, 60).

David Strachan proposed the hygiene hypothesis in 1989 (61) and was further substantiated and extended by studies. Due to the importance of bacterial colonization in healthy intestinal mucosal development, differentiation, function, and regulation, decreased microbial exposure and subsequent Th1 response stimulation in children may contribute to the development of abnormal immune responses (62). These circumstances will ultimately increase their susceptibility to allergy, asthma, and autoimmune diseases (63). Soon after birth, a vast microbiome colonizes the infant’s gut through a mother-infant microbial exchange, profoundly influencing the initial development and maturation of the neonatal microbiome (64). Common perinatal interventions, including delivery mode (cesarean-section delivery or vaginal delivery), antibiotic use, and feeding pattern (formula feeding or breastfeeding), may also contribute to the infant microbiome since maternal microbiota could transfer during pregnancy and breastfeeding. A study that orally administered a labeled *Enterococcus faecium* strain to pregnant mice showed low levels of the labeled strain transferred to the fetal intestine and a higher level transferred to the mammary glands (56). The allergic status of the mother could derange maternal counts of *Bifidobacteria*, the dominant bacteria present in breastmilk, which impacts the infant’s fecal *Bifidobacterium* levels (65). In addition, human milk oligosaccharides (HMO), which serve as prebiotics: a food source for beneficial bacteria residing in the gut, plays numerous roles in the infant’s gut (66). HMOs are protective against specific harmful pathogens due to their antimicrobial properties and preventing bacterial adhesion to the intestinal epithelium. HMOs also directly affects the epithelial and immune cells, which may subsequently affect the gut microbial composition. HMOs are produced in large diversity and abundance in human milk. These glycans are not digestible by the infant and appear to serve various functions, including prebiotic stimulation of growth of specific bacterial species. Many *Bifidobacteria* and *Bacteroides* species can transport and consume HMOs, whereas *Enterobacteriaceae* can consume non-HMOs, such as GOS and maltodextrin, but not intact HMOs. HMOs could influence the initial colonization of the infant’s gut with *Bifidobacterium* and *Bacteroides* and the distribution of the infant’s microbiome, further affecting later life (67). A careful characterization of changes in the fecal microbiota of the term infant and the ingested HMOs that pass through the intestinal tract to later appear in the infant feces confirms that, as the numbers of *Bifidobacteria* and *Bacteroides* increase in the feces, the amounts of fecal HMOs decrease, suggesting that HMOs play a significant role in shaping the microbiota of the breastfed infant (68).

In theory, fetal intestines may be exposed to commensal microbes and their products in swallowed amniotic fluid, which may therefore be an important contributor to early immune development (69). Memory CD4+ and CD8+ T cells identified towards the end of the first trimester in the human fetal gut suggested that early fetal exposure to microbial antigens may impact immunity (70). While it is not clear what the relative contribution of maternal versus fetal microbiome is to offspring immunity in response to food allergy, it is plausible that both this microbiota is critical in programming fetal immunity prior to delivery.

Despite these observations, the mechanisms underlying maternal microbiota transfer to the fetus and breastmilk remain unclear. One theory proposed that maternal dendritic cells in the Peyer’s Patch can cross the paracellular space of the intestinal epithelium to take up bacteria directly from the intestinal lumen (71). If so, this approach is advantageous because dendritic cells are relatively ineffective at killing internalized organisms. Thus, they serve to help viable bacteria reach the mammary glands and placenta (72). Once in the blood circulation, maternal-derived bacteria may be transferred to the fetus *via* the paracellular pathway of the placental barrier (71).

## Prevention and Management

**Table 1** listed the prevention and management of food allergy during prenatal and postpartum.

**Table 1:** Prevention and management of food allergy.

Prenatal prevention	
Identify High-Risk Infants	Index: IgE levels, food-specific IgE, lower-interferon/interleukin-4 ratio, and elevated levels of inflammatory cells (73–75)
	A careful assessment through questionnaires about the family history of both parents (35, 76)
Probiotic Intervention	*Lactobacillus GG*, *L. acidophilus La-5 and Bifidobacterium animalis subsp. lactis Bb-12* with inconsistent results (77, 78)
**Postpartum management**	
Immunotherapy	Oral immunotherapy, subcutaneous and subcutaneous immunotherapy (79–81)
Hydrolysate Formula	Hydrolyzed formula milk with inconsistent results (82–84)

## Identification of High-Risk Infants

Without a doubt, the identification of high-risk newborns and infants can help provide direct preventive measures. Several researchers have suggested genetic linkage markers and immunologic factors, such as elevated cord blood and infancy IgE levels, food-specific IgE, lower-interferon/interleukin-4 ratio, and elevated levels of inflammatory cells (e.g., peripheral blood and nasal eosinophils and basophils) are associated with subsequent food allergy development, which may be useful in the diagnosis of high-risk infants (73–75). However, the pattern and threshold of an immune response to food allergens vary from individual to individual. None of the symptoms related to immunologically or non-immunologically mediated allergies are pathognomonic. However, inadequate sensitivity and low predictive power limit the effectiveness of diagnostic tests, deeming them unpractical for general population screening. Instead, a careful assessment through questionnaires about the family history of both parents may be an adequate solution for large-scale screening. The American Academy of Pediatrics and several European committees and organizations recommend using atopic family history to identify high-risk infants and administer appropriate interventions for allergy prevention. A documented parental or relative food allergy would increase the likelihood of food allergy development in the offspring (35). For example, research has shown that a child can experience as high as a 7-fold increase in peanut allergy risk if he or she has a parent or sibling with the same condition (85). Therefore, infants with at least one first-degree relative (parent or sibling) with the documented allergic disease would be identified as high-risk infants (76).

## Probiotic Intervention

In the past decade, immunomodulation has been explored as a means of atopy prevention as studies have demonstrated the role of intestinal microbiota in immunity maturation during early childhood (86). It is becoming increasingly evident that intestinal microbiota influences the development of both Th1- and Th2-mediated diseases through regulatory cells and pathways, which could potentially help prevent disease-associated aberrant immune responses if adequately used for interventions (87). Several epidemiologic studies have clearly shown that reduced microbial exposure in children due to the westernized lifestyle is at the root of the increased allergy prevalence in developed countries within the last several years (“Hygiene Hypothesis”), providing further evidence regarding the role of the intestinal microbiota in allergy (88, 89).

In terms of intervention options, the use of probiotic therapy, which involves the use of living microorganisms in adequate amounts to help confer a health benefit on the host (90), has received increasing amounts of scientific documentation and is being evaluated as a means to encourage a Th1-predominant immune system. Therefore, an appropriate microbial stimulation following birth may prove to be essential in creating a balanced Th1/Th2 response of the immune system after skewing towards Th2-type immunity at birth (89, 91, 92).

*Lactobacilli* and *Bifidobacteria* inhabit the intestinal flora of non-allergic infants, while allergic infants demonstrate a different microbiota composition composed of elevated levels of *Coliforms* and *Staphylococcus aureus* (93–95). A study from Finland reported positive preventative effects of *Lactobacillus GG* against early atopic disease in high-risk children when administered prenatally to mothers (who had at least one first-degree relative (or partner) with atopic eczema, allergic rhinitis, or asthma) and postnatally for six months to their infants (88). Furthermore, maternal probiotic (a combination of *Lactobacillus rhamnosos GG, L. acidophilus La-5 and Bifidobacterium animalis subsp. lactis Bb-12*) may be sufficient for long term reduction in the cumulative incidence of offspring’s atopic dermatitis (6 year follow up) (96). Prenatal and postnatal administration of high doses of *Lactobacillus rhamnosus* GG seems to be the most promising approach for food allergy prevention in offspring (77). The studies mentioned above suggest that gastrointestinal microbiota promotes antiallergenic processes through 1 or a combination of the following ways: (1) inducing immune deviation towards a Th1-type of immune (93). (2) resulting in multiple mechanisms to increase TGF-β levels, which include microbiota-induced TGF-β production as well as enhancement of active and hence functional TGF-β levels (97). TGF-β plays an essential role in the suppression of T-helper-2-induced allergic inflammation and induction of oral tolerance (98). and (3) IgA production, an essential component of mucosal immune defense. Bacterial-derived antigens and short-chain fatty acids directly interact with B cells to induce IgA production through G-protein coupled receptors signaling, and possibly act indirectly through intestinal epithelial cells or dendritic cells in T-cell-dependent and -independent pathways (99). However, other studies reported conflicting results. The non-selected mothers received probiotic milk containing *Lactobacillus rhamnosus GG, L. acidophilus La-5*, and *Bifidobacterium animalis subsp. lactis Bb-12* from 36 weeks of gestation to 3 months postnatal during breastfeeding showed no effect on atopic sensitization during the child’s first two years of age (78). The issue when making comparisons between studies is the vast number of variables (e.g., specific species, dosage, survival rate, duration of consumption, administered age) to take into consideration before drawing any conclusions regarding the effectiveness of probiotics on the prevention of childhood food allergies.

## Immunotherapy Intervention

Various forms of immunotherapy, including oral immunotherapy (OIT) and sublingual immunotherapy (SLIT), are being more frequently used since both modalities have proven to be promising and effective in children with IgE-mediated, challenge-proven peanut allergy (80, 100–102). OIT involves the daily ingestion of the target allergen mixed with a food vehicle in gradually increasing doses (varying from milligrams to grams), while SLIT involves the administration of small amounts, on the scale of micrograms to milligrams, of allergen extract under the tongue. Early allergen exposure in small, controlled dosages may build tolerance and reduce adverse reactions in children with severe peanut allergies (47). A double-blind, randomized, placebo-controlled trial demonstrated that low-dose OIT is a safe and efficacious treatment option for children with IgE-mediated, challenge-proven peanut allergy (79).

Although oral immunotherapy appeared far more effective than subcutaneous immunotherapy (SCIT) as a treatment of peanut allergy, it carries a higher risk for adverse reactions, presumably due to the higher doses it utilizes when compared to SLIT (81, 102). Although pretreatment with SCIT before OIT led to a dramatic reduction in overall adverse events, it did not eliminate the risk of intolerable gastrointestinal symptoms, ultimately leading to the discontinuation of therapy. Therefore, it is necessary to conduct additional studies to maximize both the efficacy and tolerability of these treatment options while taking into consideration the potential use of adjuvants, modified allergens, and more extended periods of maintenance dosing.

## Hydrolysate Formula Intervention

When breastfeeding is insufficient, partially hydrolyzed, or extensively hydrolyzed, formula milk is recommended as a substitute for the first four to six months of infancy to reduce early allergen exposure in those at risk for allergic diseases (103). Some researchers found that allergy prevention in the first year of life in infants with a familial risk for atopy is possible through the administration of a hydrolyzed formula milk instead of cow’s milk formula as a supplement or substitute for breast milk (82, 104, 105). However, various recent studies do not recommend partially hydrolyzed whey or soy-based formula milk as a preventative measure against allergy or food intolerance in high-risk infants (83, 84, 106). To date, evidence regarding this matter remains inconclusive. Studies have findings that support both sides of the discussion on whether hydrolysate formula is an appropriate preventative measure against food allergy development in at-risk infants. A conclusive judgment should consider various factors, such as different inclusion criteria, insufficient randomization, blinding, and poorly defined diagnostic criteria.

## Summary

The prevalence of food allergies has increased among infants and children, and its proneness is primarily influenced by maternal immunity, microbiota, breastfeeding, allergy exposure and genetic factors. The large-scale population identification of high-risk infants and probiotic supplementation may help provide good prenatal intervention options. Although current postpartum management such as immunotherapy and hydrolysate formula may be advised, the scientific results from published studies remain inconsistent, limiting our ability to draw firm conclusions. With that in mind, additional research is required to further elucidate the maternal influences of proneness to develop food allergy in offspring.

## Author Contributions

The review was written and edited by LJ. C-WS and HS helped supervise the project. All authors provided critical feedback and helped shape the final review.

## Funding

This work was supported by grants from the National Institutes of Health-R21 AI121997 (to HS), R21 AI144738-01A1 (to CWS) and by the Nutrition Obesity Research Center at Harvard (P30 DK040561). Chien-wen Su was supported by a Pilot Feasibility Grant from the Nutrition Obesity Research Center at Harvard (P30 DK040561). Lefei Jiao was sponsored by the China Scholarship Council and Ningbo Public Welfare Science and Technology Project (202002N3041). The funders had no role in study design, collection, analysis, or interpretation of data.

## Conflict of Interest

The authors declare that the research was conducted in the absence of any commercial or financial relationships that could be construed as a potential conflict of interest.

## Publisher’s Note

All claims expressed in this article are solely those of the authors and do not necessarily represent those of their affiliated organizations, or those of the publisher, the editors and the reviewers. Any product that may be evaluated in this article, or claim that may be made by its manufacturer, is not guaranteed or endorsed by the publisher.
